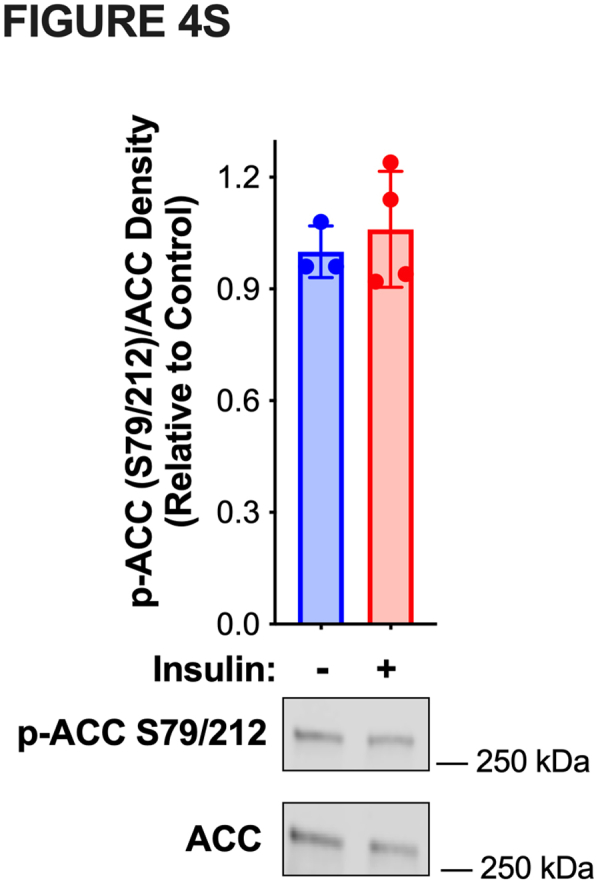# Reciprocal regulation of cardiac β-oxidation and pyruvate dehydrogenase by insulin

**DOI:** 10.1016/j.jbc.2024.107412

**Published:** 2024-05-23

**Authors:** Abdallah Elnwasany, Heba A. Ewida, Ivan Menendez-Montes, Monika Mizerska, Xiaorong Fu, Chai-Wan Kim, Jay D. Horton, Shawn C. Burgess, Beverly A. Rothermel, Pamela A. Szweda, Luke I. Szweda

**Affiliations:** 1 Division of Cardiology, Department of Internal Medicine, University of Texas Southwestern Medical Center, Dallas, Texas, USA; 2 Department of Pharmaceutical Sciences, Jerry H. Hodge School of Pharmacy, Texas Tech University Health Sciences Center, Amarillo, Texas, USA; 3 Faculty of Pharmacy, Future University in Egypt (FUE), Cairo, Egypt; 4 Department of Pharmacology, Center for Human Nutrition, University of Texas Southwestern Medical Center, Dallas, Texas, USA; 5 Departments of Internal Medicine and Molecular Genetics, Center for Human Nutrition, University of Texas Southwestern Medical Center, Dallas, Texas, USA

**Keywords:** heart, mitochondria, insulin, pyruvate dehydrogenase, acetyl-CoA carboxylase, malonyl-CoA, β-oxidation

## Abstract

The heart alters the rate and relative oxidation of fatty acids and glucose based on availability and energetic demand. Insulin plays a crucial role in this process diminishing fatty acid and increasing glucose oxidation when glucose availability increases. Loss of insulin sensitivity and metabolic flexibility can result in cardiovascular disease. It is therefore important to identify mechanisms by which insulin regulates substrate utilization in the heart. Mitochondrial pyruvate dehydrogenase (PDH) is the key regulatory site for the oxidation of glucose for ATP production. Nevertheless, the impact of insulin on PDH activity has not been fully delineated, particularly in the heart. We sought *in vivo* evidence that insulin stimulates cardiac PDH and that this process is driven by the inhibition of fatty acid oxidation. Mice injected with insulin exhibited dephosphorylation and activation of cardiac PDH. This was accompanied by an increase in the content of malonyl-CoA, an inhibitor of carnitine palmitoyltransferase 1 (CPT1), and, thus, mitochondrial import of fatty acids. Administration of the CPT1 inhibitor oxfenicine was sufficient to activate PDH. Malonyl-CoA is produced by acetyl-CoA carboxylase (ACC). Pharmacologic inhibition or knockout of cardiac ACC diminished insulin-dependent production of malonyl-CoA and activation of PDH. Finally, circulating insulin and cardiac glucose utilization exhibit daily rhythms reflective of nutritional status. We demonstrate that time-of-day-dependent changes in PDH activity are mediated, in part, by ACC-dependent production of malonyl-CoA. Thus, by inhibiting fatty acid oxidation, insulin reciprocally activates PDH. These studies identify potential molecular targets to promote cardiac glucose oxidation and treat heart disease.

The heart relies primarily on the oxidation of fatty acids and glucose to produce ATP. The ability to alter the rate and relative use of fatty acids and glucose allows the heart to adapt to changes in nutrient status and energetic demand. Insulin plays a central role in this process decreasing fatty acid and increasing glucose oxidation in the heart when glucose availability is elevated (1, 2). Given that insulin resistance and diabetes are independent risk factors for cardiovascular disease (3, 4, 5, 6, 7), abnormalities in fatty acid and glucose oxidation are widely believed to play a causal role in pathogenesis. Nevertheless, the impact of insulin on key regulatory components of fatty acid and glucose oxidation in the heart is not known. Gaps in mechanistic information prevent the identification of specific defects in insulin signaling that occur during the progression of heart disease and the impact on cardiac metabolism and function. As such, targeted metabolic interventions are limited.

The primary sites for the regulation of fatty acid and glucose oxidation are mitochondrial carnitine palmitoyltransferase 1 (CPT1) and pyruvate dehydrogenase (PDH), respectively. CPT1 catalyzes the conversion of acyl-CoA to acyl-carnitine providing transport of fatty acids into the mitochondria for oxidation. CPT1 is inhibited by malonyl-CoA produced by acetyl-CoA carboxylase (ACC) (8, 9, 10, 11). PDH catalyzes the decarboxylation of pyruvate to acetyl-CoA and the reduction of NAD^+^ to NADH, committing glycolysis-derived pyruvate to the majority of ATP generated from the oxidation of glucose. A multimeric enzyme complex composed of E1α, E1β, E2, and E3 subunits, PDH is inhibited upon phosphorylation of the E1α subunit by pyruvate dehydrogenase kinases (PDK-1, -2, and -4, isozymes in the heart) and activated upon dephosphorylation by pyruvate dehydrogenase phosphatases (PDP-1 and -2) (12, 13, 14, 15). Insulin is known to increase the production of malonyl-CoA (16, 17, 18, 19, 20) or activate PDH (21, 22, 23, 24) consistent with decreasing fatty acid and increasing glucose oxidation. However, these findings are primarily from investigations of the effects of insulin on adipose tissue, liver, and/or skeletal muscle metabolism. While these tissues are metabolically distinct, similar modes of regulation are often inferred for the heart. There are a limited number of studies on the effects of insulin on cardiac malonyl-CoA content or PDH activity in fed or non-diabetic animals. In hearts from mice fed a control diet and perfused *ex vivo* with glucose and palmitate, insulin has been reported to increase ACC activity (25) and, in a separate study, malonyl-CoA content (16). More recently, using a similar *ex vivo* heart model, insulin-induced increases in glucose oxidation were accompanied by dephosphorylation of PDH consistent with the activation of PDH (1). However, PDH activity was not measured (1), and the mechanisms by which insulin induces an increase in malonyl-CoA and dephosphorylation of PDH, particularly in the heart, have not been defined.

In the current study, we sought evidence for the *in vivo* effects of insulin on cardiac PDH activity and malonyl-CoA content. Furthermore, we tested whether the activation of PDH and ACC-dependent production of malonyl-CoA are integrally linked. Hearts were analyzed from mice administered insulin and pharmacologic inhibitors of CPT1 and ACC and from mice with inducible cardiomyocyte-specific double knockout of ACC1/2. PDH phosphorylation status and activity were measured in concert with the cardiac content of targeted metabolites to gain mechanistic insight into insulin-dependent regulation of fatty acid and pyruvate oxidation. We present *in vivo* evidence that, in the heart, ACC-dependent increases in malonyl-CoA and inhibition of fatty acid oxidation underlie reciprocal activation PDH upon administration of insulin. The physiological significance of these findings is exemplified by results indicating that intrinsic changes in PDH activity at times of day marked by distinct differences in nutritional status and circulating insulin (26, 27) are, in part, dependent on changes in malonyl-CoA content.

## Results

### Insulin activates pyruvate dehydrogenase and induces increases in the content of malonyl-CoA, acetyl-CoA, and CoASH in the heart

**Figure 1:**
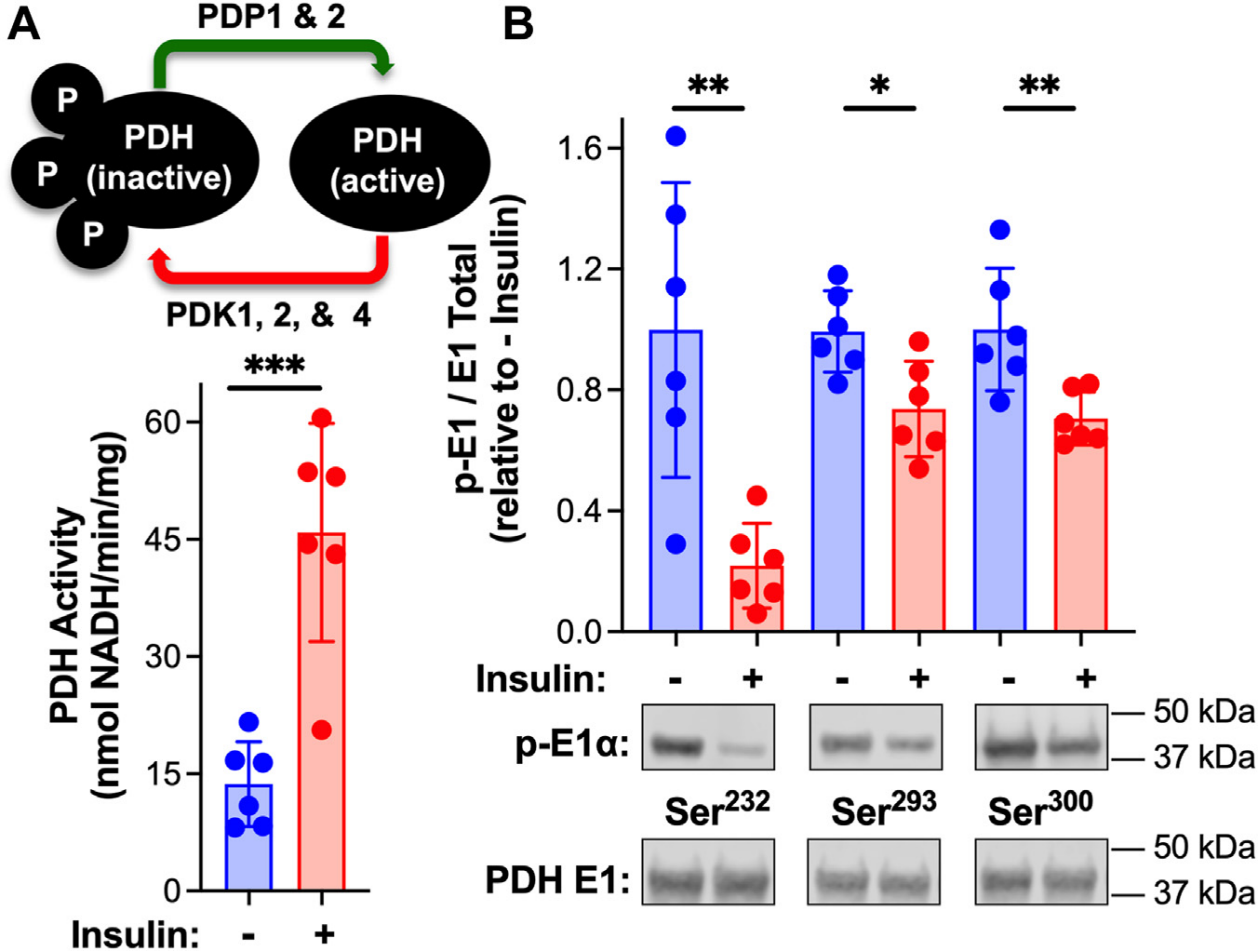
**Insulin-induced activation of cardiac pyruvate dehydrogenase.** C57BL/6N male mice received an intraperitoneal injection of saline or insulin (0.01 U/g body weight) at 8 AM. At 15 min post-injection hearts were excised, homogenized, and mitochondria isolated (+DCA and NaF). *A*, PDH activity was measured spectrophotometrically and (*B*) E1α subunit content and phosphorylation status of each site on the PDH E1α subunit (Ser^232^, Ser^293^, and Ser^300^) were measured by Western blot followed by densitometric analysis. Values are presented as the mean ± SD (n = 6) where significant differences (2-tailed *t* test) are indicated by ∗*p* < 0.05, ∗∗*p* < 0.01, and ∗∗∗*p* < 0.001. Each data point indicates a separate animal.

Injection of mice with insulin resulted in a 3-fold increase in cardiac PDH activity (Fig. 1*A*). The increase in activity was accompanied by dephosphorylation at each of the 3 phosphorylation sites on the E1α subunit of PDH (Fig. 1*B*). Serine 232 exhibited the greatest decrease in phosphorylation in response to insulin. Metabolites that serve as substrates, products, and regulators of β-oxidation and PDH were quantified in extracts of frozen cardiac tissue. We discovered that insulin induced a decrease in pyruvate content and increases in the levels of malonyl-CoA, acetyl-CoA, and CoASH in the heart (Table 1). No significant changes were observed in the content of NADH, NAD^+^, ATP, ADP, or AMP, the ratios of acetyl-CoA/CoASH and NAD^+^/NADH, and energy charge (Table 1). Diminished pyruvate is in keeping with enhanced PDH activity and pyruvate oxidation (Fig. 1). Malonyl-CoA is an inhibitor of carnitine palmitoyltransferase I (CPT1) and import of fatty acids into the mitochondria for oxidation (10). Thus, increased cardiac malonyl-CoA content is consistent with inhibition of β-oxidation by insulin. Finally, mitochondrial β-oxidation and PDH have in common the use of CoASH to produce acetyl-CoA. It is therefore interesting that insulin-induced an increase in both CoASH and acetyl-CoA. Given the concomitant increase in cardiac PDH activity and malonyl-CoA content, we sought to determine whether: (1) Inhibition of β-oxidation is sufficient to activate PDH and (2) Production of malonyl-CoA is required for stimulation of PDH activity by insulin.

**Table 1:** Insulin-induced changes in cardiac metabolites

Metabolite	Control	Insulin	*p*-value (*t* test)
	Units: (nmol/mg protein ± standard deviation)		
Pyruvate	3.90 ± 0.66	3.05 ± 0.49	0.02
Malonyl-CoA	0.029 ± 0.006	0.043 ± 0.002	0.0006
Acetyl-CoA	0.17 ± 0.05	0.30 ± 0.01	0.0002
CoASH	0.18 ± 0.07	0.37 ± 0.05	0.0004
NADH	1.31 ± 0.25	1.31 ± 0.25	Not Significant
NAD^+^	12.1 ± 2.3	14.3 ± 0.9	Not Significant
ATP	20.4 ± 4.0	24.2 ± 3.7	Not Significant
ADP	24.4 ± 3.4	25.1 ± 2.9	Not Significant
AMP	12.9 ± 2.7	11.5 ± 3.3	Not Significant
Ratios			
Acetyl-CoA/CoASH	1.03 ± 0.36	0.81 ± 0.11	Not Significant
NAD^+^/NADH	9.7 ± 3.5	11.3 ± 2.9	Not Significant
Energy charge	0.56 ± 0.05	0.61 ± 0.04	Not Significant

Male C57BL/6N mice received an intraperitoneal injection of saline or insulin (0.01 U/g body weight) at 8 AM. Hearts were excised and frozen 15 min post-injection. Heart tissue was pulverized in liquid nitrogen, extracted, and metabolites resolved and levels quantified by LC- and GC-mass spectrometry. Values are presented as the mean ± SD (n = 6 separate animals) with *p*-value (2-tailed *t* test) as indicated.

### Inhibition of β-oxidation induces an increase in cardiac pyruvate dehydrogenase activity

**Figure 2:**
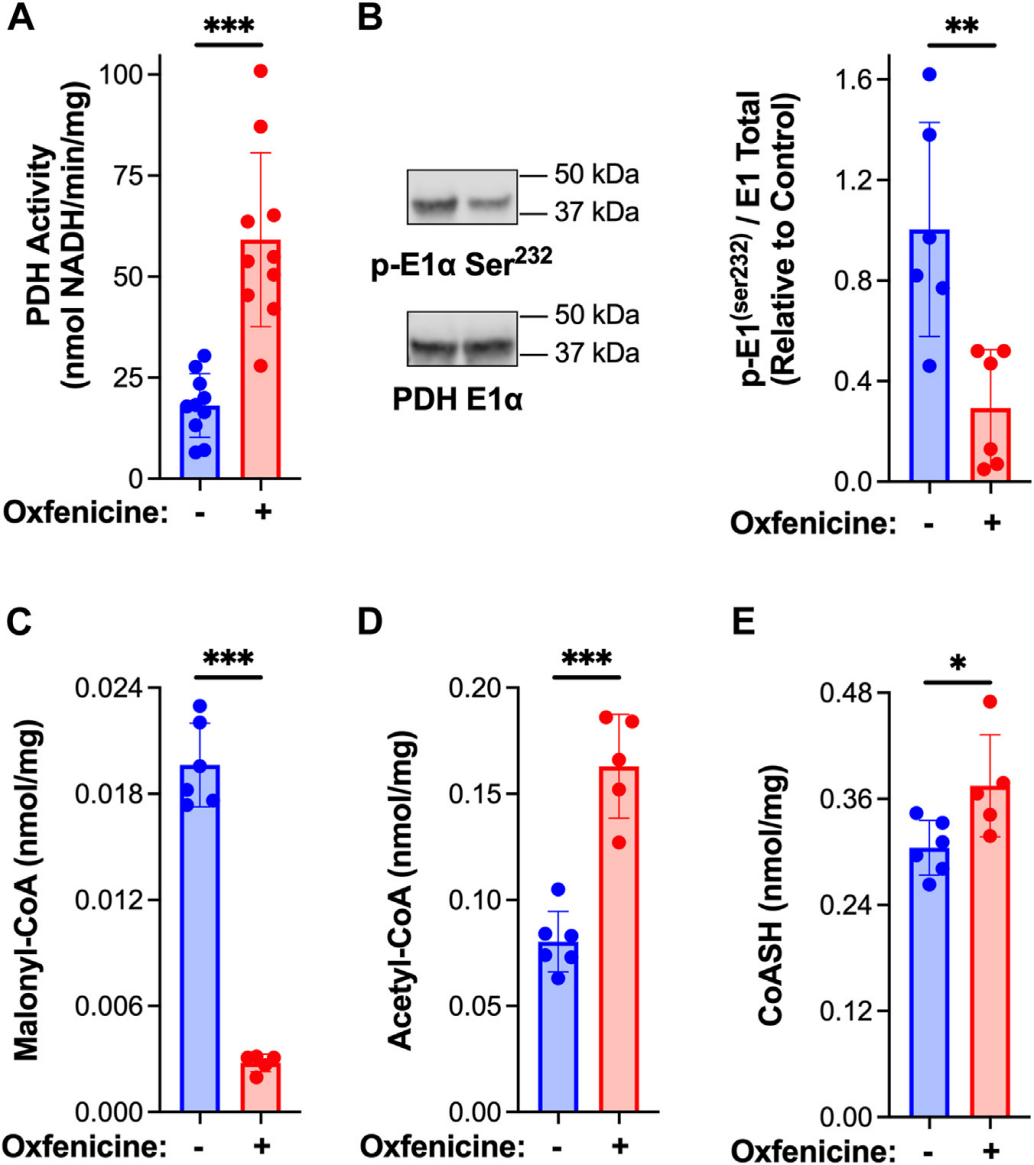
**Inhibition of β-oxidation activates pyruvate dehydrogenase and increases CoASH and acetyl-CoA content in the heart.** Male C57BL/6N mice received an intraperitoneal injection of saline or oxfenicine (0.15 mg/g) at 12 PM. Hearts were excised 30 min post-injection, homogenized, and mitochondria were isolated (+DCA and NaF). *A*, PDH activity was measured spectrophotometrically. *B*, E1α subunit content and phosphorylation status of Ser^232^ on the PDH E1α subunit were measured by Western blot followed by densitometric analysis. Cardiac tissue excised and frozen 30 min post-oxfenicine injection was extracted, metabolites resolved by reverse phase HPLC and (*C*) malonyl-CoA, (*D*) acetyl-CoA, and (*E*) CoASH quantified by UV/Vis spectroscopy. Values are presented as the mean ± SD (n = 5–9) where significant differences (2-tailed *t* test) are indicated by ∗*p* < 0.05, ∗∗*p* < 0.01, and ∗∗∗*p* < 0.001. Each data point indicates a separate animal.

To determine whether inhibition of mitochondrial fatty acid transport and β-oxidation activates cardiac PDH, mice were injected with the CPT1 inhibitor, oxfenicine (28). Treatment with oxfenicine (30 min) resulted in dephosphorylation and activation of PDH in the heart (Fig. 2, *A* and *B*). In contrast to insulin, oxfenicine induced a decrease in malonyl-CoA levels, suggestive of a compensatory response to suppressed β-oxidation (Fig. 2*C*). Thus, pharmacologic inhibition of CPT1 and β-oxidation is sufficient to activate cardiac PDH. Further, as observed with insulin administration, the levels of cardiac acetyl-CoA and CoASH increased upon injection of oxfenicine (Fig. 2, *D* and *E*). Therefore, acute inhibition of β-oxidation and activation of PDH result in increases in acetyl-CoA and CoASH content in the heart.

### Acetyl-CoA carboxylase is required for the activation of cardiac pyruvate dehydrogenase by insulin

**Figure 3:**
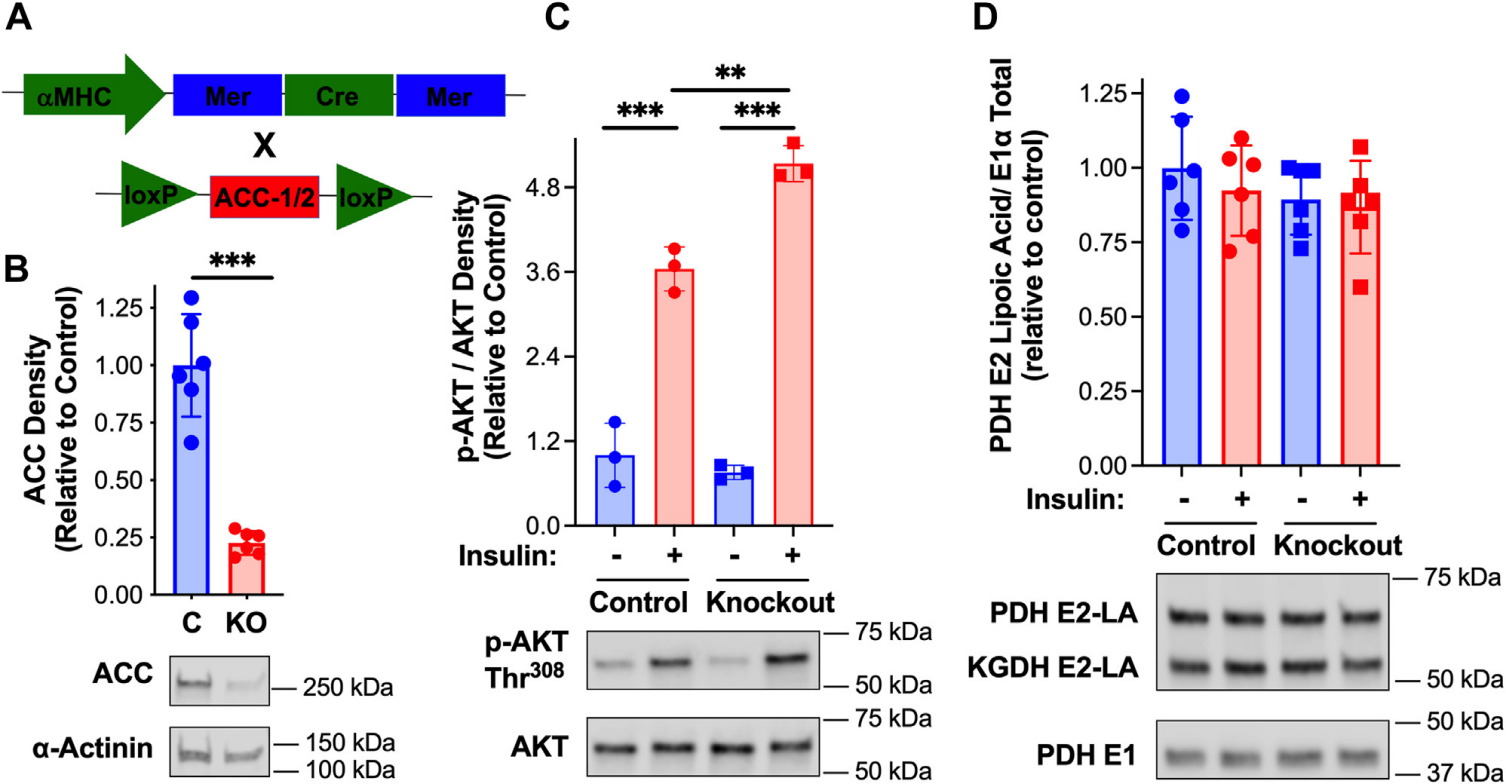
**Inducible cardiomyocyte-specific acetyl-CoA carboxylase-1/2 knockout mice.***A*, inducible cardiomyocyte-specific ACC-1/2 knockout mice (αMHC-MerCreMer cross with *Acc1*^f/f^/*Acc2*^f/f^) were generated as indicated. *B*, three days following the final tamoxifen injection (3 injections every other day of 0.5 mg in 50 μl 10% v/v ethanol/sesame oil) hearts were harvested from control and ACC-1/2 knockout mice and total cardiac ACC protein quantified by Western blot followed by densitometric analysis (n = 6). *C*, control, and ACC KO mice were injected with insulin (0.05 U/g) intraperitoneally at 8 AM. Hearts were then excised and frozen 10 min post-injection. Heart homogenate was centrifuged (500*g*, 5 min, 4 °C) and the supernatant (S1) evaluated for (*C*) total AKT and phosphorylation status of the Thr^308^ site on AKT and (*D*) lipoic acid conjugated to PDH E2 or α-ketoglutarate dehydrogenase (KGDH) E2 and total PDH E1 by Western blot analysis followed by densitometric analysis. Values are presented as the mean ± SD (n = 3–6) where significant differences (2-tailed *t* test or in experiments with multiple variables by ANOVA with the Tukey test) are indicated by ∗*p* < 0.05, ∗∗*p* < 0.01, and ∗∗∗*p* < 0.001. Each data point indicates a separate animal.

**Figure 4:**
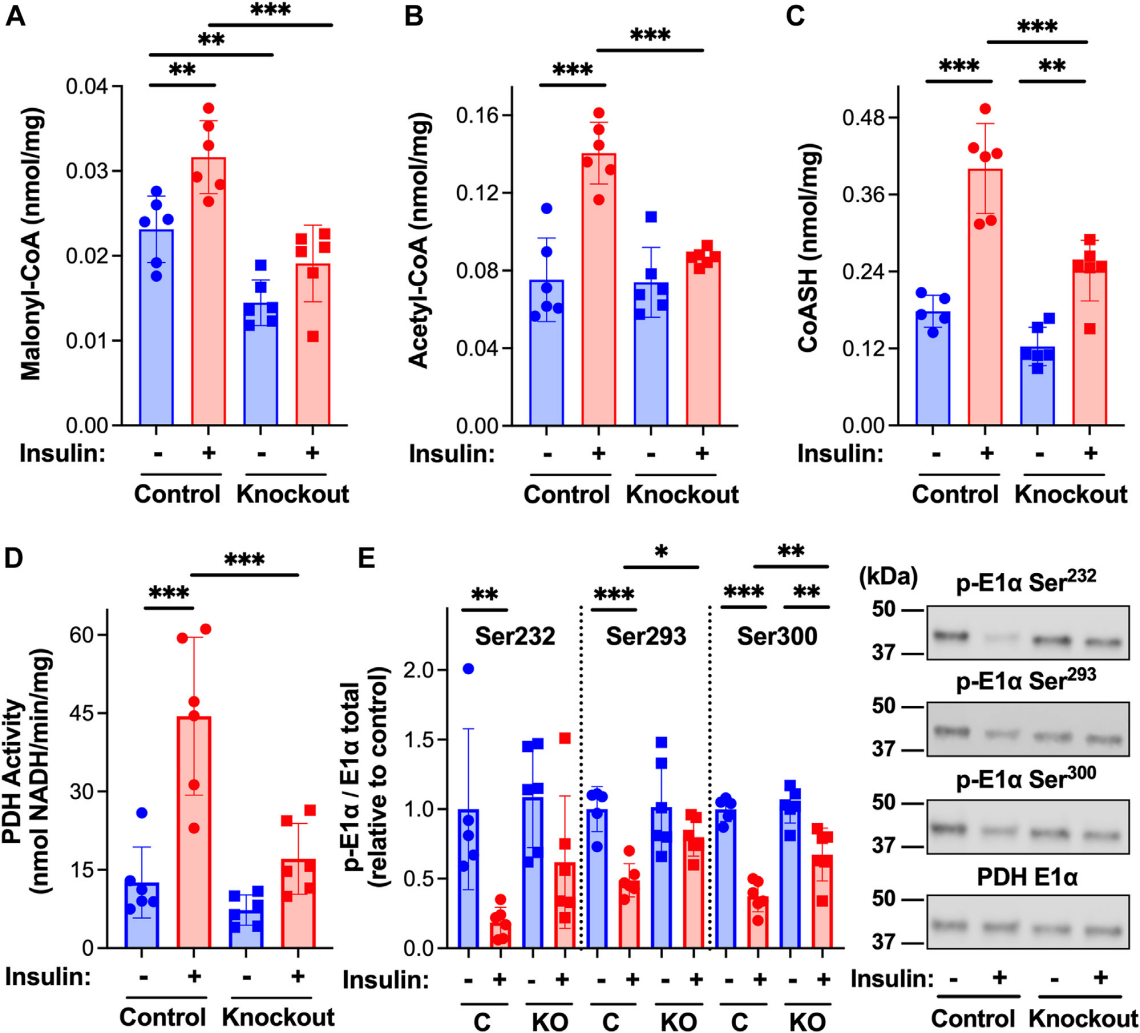
**Cardiomyocyte-specific deletion of acetyl-CoA carboxylase reduces insulin-dependent increases in malonyl-CoA and pyruvate dehydrogenase activation.** Control (*Acc1*^f/f^/*Acc2*^f/f^ and αMHC-MerCreMer) and ACC-1/2 knockout mice, 3 days post-tamoxifen, received an intraperitoneal injection of saline or insulin (0.05 U/g body weight) at 8 AM. Hearts were excised 10 min post-injection, a piece of the *left* ventricle was frozen for metabolite analysis, and the remainder was homogenized and mitochondria isolated (+DCA and NaF). Frozen tissue from control and ACC knockout ± insulin was extracted, and metabolites resolved by reverse phase HPLC and (*A*) malonyl-CoA, (*B*) acetyl-CoA, and (*C*) CoASH quantified by UV/Vis spectroscopy. Isolated mitochondria were evaluated for (*D*) PDH activity as measured spectrophotometrically and (*E*) E1α subunit content and phosphorylation status of each site on the PDH E1α subunit (Ser^232^, Ser^293^, and Ser^300^) by Western blot followed by densitometric analysis. Values are presented as the mean ± SD (n = 6) where significant differences (ANOVA with the Tukey test) are indicated by ∗*p* < 0.05, ∗∗*p* < 0.01, and ∗∗∗*p* < 0.001. Each data point indicates a separate animal.

**Supplemental Figure S1:**
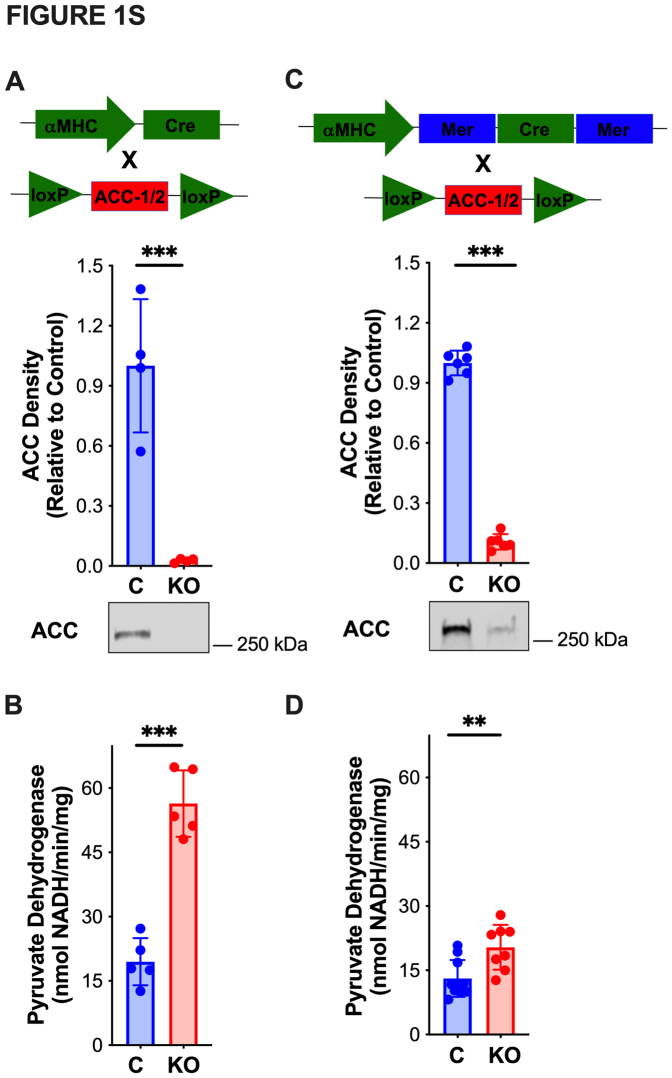


Acetyl-CoA carboxylase (ACC) catalyzes the production of malonyl-CoA (8, 9, 11). To determine if activation of PDH by insulin (Fig. 1) requires malonyl-CoA production (Table 1), cardiomyocyte-specific ACC knockout mice were utilized. Constitutive deletion of *Acc1* and *Acc2* alleles specifically in cardiomyocytes resulted in a basal increase in PDH activity, perhaps to offset sustained increases in β-oxidation (Fig. S1, *A* and *B*). We, therefore, generated and characterized inducible cardiomyocyte-specific *Acc1*/*Acc2* knockout mice (Fig. 3*A*). Analyses of hearts of αMHC-MerCreMer x *Acc1*^f/f^/*Acc2*^f/f^ mice 4 weeks post-tamoxifen, revealed a ∼90% reduction in ACC1/2 protein content (Fig. S1*C*). However, an increase in PDH activity was observed (Fig. S1*D*). In contrast, 3 days following the final tamoxifen injection no change in PDH activity relative to control mice was evident (Fig. 4*D*). Importantly, the cardiac content of ACC1/2 was ∼75% reduced compared to control mice (Fig. 3*B*). ACC1/2 knockout 3 days post-tamoxifen did not diminish insulin signaling upstream of AKT. In fact, insulin-dependent AKT phosphorylation was increased in ACC1/2 knockout mice (Fig. 3*C*). Additionally, levels of lipoic acid conjugated to the PDH E2 subunit were unchanged in hearts of ACC1/2 knockout relative to control mice (Fig. 3*D*). We therefore chose to perform insulin injections followed by biochemical analyses in knockout (αMHC-MerCreMer x *Acc1*^f/f^/*Acc2*^f/f^) and control (*Acc1*^f/f^/*Acc2*^f/f^ and αMHC-MerCreMer) mice 3 days post-tamoxifen.

Inducible cardiomyocyte-specific knockout of ACC1/2 reduced basal cardiac malonyl-CoA content relative to control mice (Fig. 4*A*). Insulin induced a significant increase in malonyl-CoA in control but not ACC1/2 knockout mice. Residual and/or non-cardiomyocyte ACC (Fig. 3*B*) is likely responsible for malonyl-CoA in the cardiomyocyte-specific knockout mice. ACC1/2 knockout reduced insulin-dependent increases in acetyl-CoA and CoASH (Fig. 4, *B* and *C*). Importantly, dephosphorylation and activation of PDH upon insulin administration were largely diminished in ACC1/2 knockout mice (Fig. 4, *D* and *E*). Collectively, these results support the conclusion that ACC-dependent production of malonyl-CoA and inhibition of β-oxidation are required for activation of PDH by insulin.

**Figure 5:**
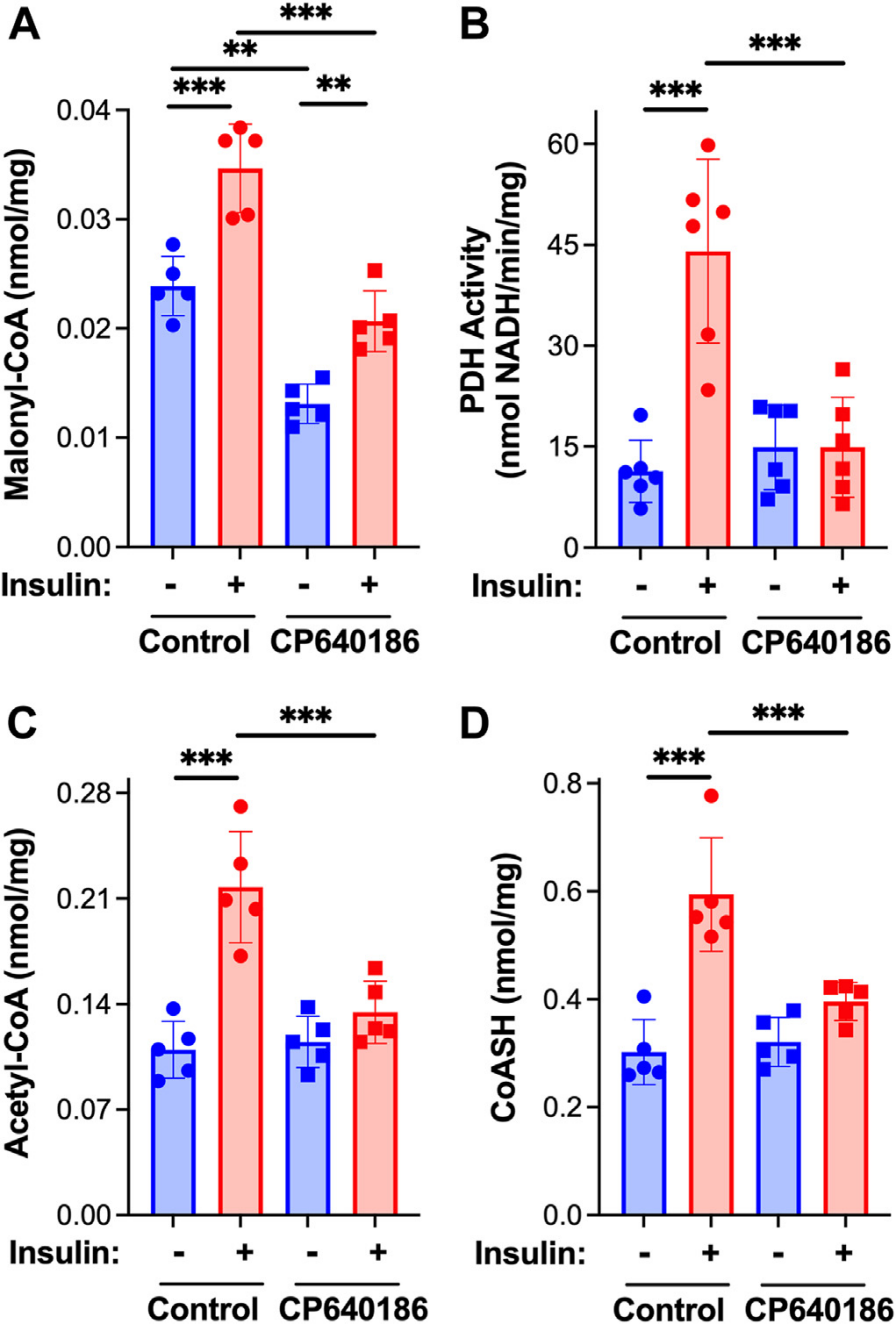
**Insulin-induced activation of cardiac pyruvate dehydrogenase is abolished by inhibition of acetyl-CoA carboxylase.** Male C57BL/6N mice were administered the ACC inhibitor, CP 640186 (0.1 mg/g in 0.5% methyl cellulose) or 0.5% methyl cellulose, by gavage (±CP) at 7:30 AM. As indicated, insulin (0.05 U/g body weight) was injected intraperitoneally 30 min later at 8 AM and 10 min after insulin injection hearts were excised, a piece of the *left* ventricle frozen for metabolite analysis, and the remainder homogenized and mitochondria isolated (+DCA and NaF). *A*, PDH activity in isolated mitochondria was measured spectrophotometrically and frozen tissue was extracted, metabolites resolved by reverse phase HPLC and (*B*) malonyl-CoA, (*C*) acetyl-CoA, and (*D*) CoASH quantified by UV/Vis spectroscopy. Values are presented as the mean ± SD (n = 6) where significant differences (ANOVA with the Tukey test) are indicated by ∗*p* < 0.05,∗∗*p* < 0.01, and∗∗∗*p* < 0.001. Each data point indicates a separate animal.

**Supplemental Figure S2:**
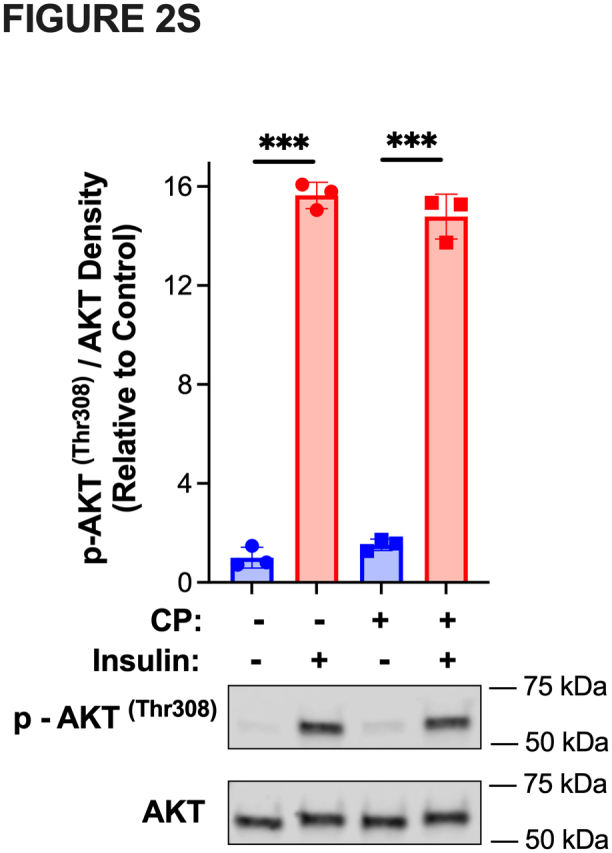


Gene ablation can induce compensatory responses, as exemplified in the constitutive and inducible cardiomyocyte-specific ACC knockout mice 4 weeks post-tamoxifen (Fig. S1). To further test the requirement for ACC and malonyl-CoA production in activation of PDH by insulin, mice were treated 30 min prior to insulin injection with the isozyme-nonselective ACC inhibitor [(3R)-1'-(9-anthracenylcarbonyl)[1,4′-bipiperidin]-3-yl]-4-morpholinyl-methanone (CP-640186) (29, 30). Insulin signaling upstream of AKT was not impacted by inhibition of ACC as judged by AKT phosphorylation (Fig. S2). Pharmacologic inhibition of ACC reduced the basal and insulin-stimulated content of malonyl-CoA in the heart (Fig. 5*A*). Activation of cardiac PDH by insulin was abolished in mice treated with the ACC inhibitor (Fig. 5*B*). Furthermore, and in keeping with results obtained with ACC-1/2 knockout mice, inhibition of ACC greatly reduced insulin-dependent increases in acetyl-CoA and CoASH (Fig. 5, *C* and *D*) indicative of lack of inhibition of β-oxidation (Fig. 2, *D* and *E*). These findings are consistent with malonyl-CoA-dependent inhibition of β-oxidation as responsible for insulin-induced activation of cardiac PDH.

### Acetyl-CoA carboxylase contributes to diurnal changes in pyruvate dehydrogenase activity in the heart

**Figure 6:**
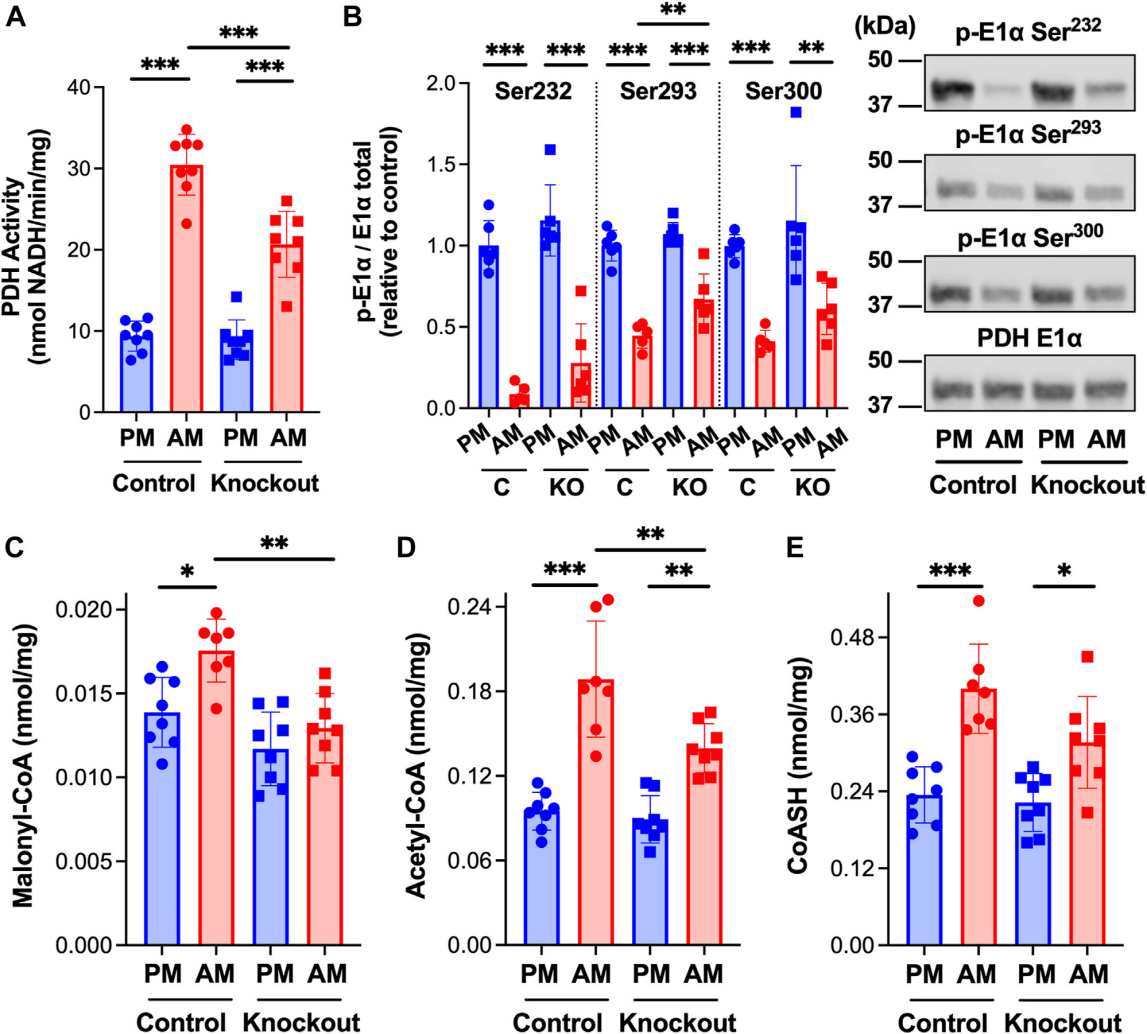
**Active phase increase in cardiac pyruvate dehydrogenase activity is mediated by acetyl-CoA carboxylase.** Three days post-tamoxifen hearts were excised and snap frozen from control (*Acc1*^f/f^/*Acc2*^f/f^) and ACC-1/2 knockout mice at 12 PM or 12 AM as indicated. Heart homogenate was prepared, (*A*) PDH activity was measured spectrophotometrically (n = 8), and (*B*) E1α subunit content and phosphorylation status of each site on the PDH E1α subunit (Ser^232^, Ser^293^, and Ser^300^) was assessed by Western blot followed by densitometric analysis (n = 6). Metabolites were extracted from frozen heart tissue and (*C*) malonyl-CoA, (*D*) acetyl-CoA, and (*E*) CoASH resolved by reverse phase HPLC and quantified by UV/Vis spectroscopy (n = 8). Values are presented as the mean ± SD where significant differences (ANOVA with the Tukey test) are indicated by ∗*p* < 0.05, ∗∗*p* < 0.01, and ∗∗∗*p* < 0.001. Each data point indicates a separate animal.

**Supplemental Figure S3:**
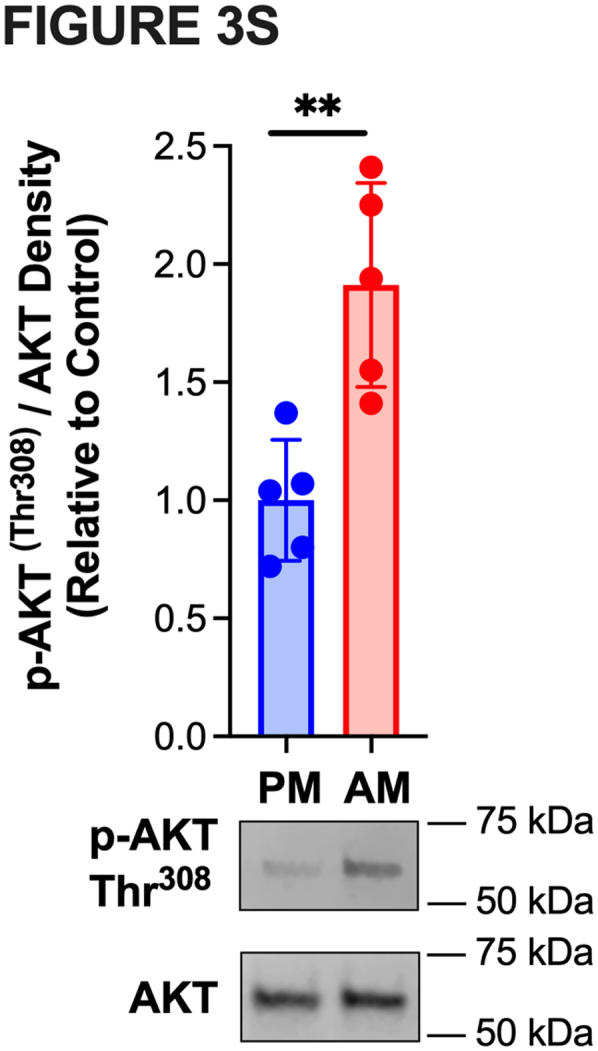


Circulating levels of insulin exhibit diurnal variations, peaking during an animal’s fed/active phase when glucose availability and utilization are enhanced (26, 27) consistent with elevated AKT phosphorylation at 12 AM relative to 12 PM (Fig. S3). If insulin-dependent inhibition of β-oxidation contributes to changes in cardiac PDH activity over the 24-h cycle, changes in myocardial PDH activity should reflect changes in malonyl-CoA content. We found that PDH activity (Fig. 6*A*) and malonyl-CoA (Fig. 6*C*) content were significantly higher in hearts excised from control mice during the active (12 AM, ZT 18) relative to inactive (12 PM, ZT 6) phase. The increase in PDH activity corresponded to reduced E1α phosphorylation of the enzyme (Fig. 6*B*). Acetyl-CoA and CoASH were higher during the active phase (Fig. 6, *D* and *E*), consistent with inhibition of β-oxidation and an increase in pyruvate relative to fatty acid oxidation. No difference in the ratio of acetyl-CoA/CoASH was observed at 12 AM compared to 12 PM. Importantly, increases in cardiac PDH activity and the content of malonyl-CoA, acetyl-CoA, and CoASH at 12 AM relative to 12 PM were diminished in cardiomyocyte-specific ACC-1/2 knockout mice. These results offer the first evidence that ACC-dependent production of malonyl-CoA and subsequent inhibition of β-oxidation are, in part, responsible for the activation of PDH during the fed/active phase.

## Discussion

The inability of the heart to respond to insulin and appropriately alter the relative oxidation of fatty acids and glucose is associated with cardiovascular disease and heart failure (3, 4, 5, 6, 7). However, mechanisms by which insulin regulates mitochondrial substrate oxidation in the heart and how perturbations in this regulation contribute to disease progression are largely unknown. Our results provide *in vivo* evidence that insulin increases cardiac PDH activity and the content of malonyl-CoA, a potent inhibitor of CPT1 (10) and, thus, mitochondrial fatty acid oxidation. Furthermore, the effects of insulin on the key regulatory sites for glucose and fatty acid oxidation are interdependent. ACC-dependent production of malonyl-CoA is required for the activation of cardiac PDH by insulin. Cardiomyocyte-specific knockout or pharmacologic inhibition of ACC was found to reduce insulin-dependent increases in malonyl-CoA and prevent the activation of PDH by insulin. The physiologic significance of these findings is evident by the concomitant suppression of time-of-day dependent increases in cardiac malonyl-CoA content and PDH activity in ACC knockout mice (Fig. 6) during the active/fed phase when circulating insulin is elevated (26, 27). Insulin-dependent inhibition of fatty acid oxidation by malonyl-CoA likely leads to the activation of PDH, as pharmacologic inhibition of CPT1 is sufficient to increase PDH activity. Our findings support the conclusion that inhibition of fatty acid oxidation and activation of PDH by insulin are directly linked in the heart. It will be critical in future studies to directly measure glucose and fatty acid oxidation *in vivo* in response to insulin and various genetic and pharmacologic perturbations employed in the current study. Technological advances, such as stable isotope labeling and hyperpolarized magnetic resonance imaging, provide the potential for *in vivo* measures that reflect physiologic regulation. The interdependence of insulin-induced increases in cardiac malonyl-CoA content and activation of PDH along with changes in the abundance of metabolites known to regulate PDKs, PDPs, and CPT1 provide the foundation for future investigations on the mechanisms by which: 1) Insulin increases cardiac malonyl-CoA levels and 2) Inhibition of β-oxidation elicits the dephosphorylation and activation of PDH.

ACC catalyzes the carboxylation of acetyl-CoA to form malonyl-CoA while MCD is responsible for decarboxylation and removal of malonyl-CoA (8, 9, 11, 31, 32, 33, 34). Thus, insulin-dependent increases in malonyl-CoA could be due to increases in ACC (25) and/or decreases in MCD activities. We have shown that knockout or inhibition of ACC reduces basal and insulin-stimulated cardiac malonyl-CoA content. These results suggest that insulin stimulates ACC-dependent production of malonyl-CoA. While ACC can be activated by dephosphorylation of serine-79 (ACC1)/serine-212 (ACC2) (35), we found no evidence for dephosphorylation of this site(s) in response to insulin (Fig. S4) despite an increase in malonyl-CoA content (Table 1 and Fig. 4*A*). Thus, other phosphorylation sites/modifications and/or mechanism(s) could be involved. Possibilities include increases in the concentration of acetyl-CoA, the substrate of ACC, allosteric activator(s) of the enzyme such as citrate and CoASH, and/or prolyl hydroxylation (8, 9, 34, 36, 37, 38). Our results demonstrate that acetyl-CoA increases in response to insulin. However, knockout or inhibition of ACC largely prevented the increase in acetyl-CoA (Figs. 4*B* and 5*C*) and administration of the CPT1 inhibitor oxfenicine (28) induced an increase in cardiac acetyl-CoA despite a drop in malonyl-CoA (Fig. 2). Thus, the increase in acetyl-CoA does not appear to be a direct response to insulin but rather a result of subsequent inhibition of β-oxidation and/or activation of PDH. Our results do not rule out a role for MCD in insulin-induced increases in malonyl-CoA. It is interesting to note that malonyl-CoA levels fell in response to pharmacologic inhibition of CPT1 (oxfenicine (28)) indicating regulation of malonyl-CoA decarboxylation by MCD. Finally, a threshold concentration of malonyl-CoA appears required for the activation of PDH. Knockout or inhibition of ACC reduced malonyl-CoA by ∼50% with no change in PDH activity while a 40 to 50% increase in malonyl-CoA in response to insulin results in a ∼3-fold increase in PDH activity. As such, potential factors that influence the binding of malonyl-CoA to CPT1 must be considered in the regulation of β-oxidation and, in turn, PDH, particularly given the high affinity of the cardiac isoform CPT1B for malonyl-CoA (10, 39). Future studies must therefore identify the mechanism(s) by which insulin increases cardiac malonyl-CoA levels and factors that influence inhibition of CPT1 by malonyl-CoA, essential components of the heart's response to changes in nutritional status.

Acetyl-CoA is a potent feedback inhibitor of PDH and activates pyruvate dehydrogenase kinases (PDK) promoting phosphorylation and inactivation of PDH. In contrast, CoASH is a required co-factor of PDH, inhibits PDK, and promotes the degradation of PDK4 by the LonP1 protease (12, 13, 15, 40, 41). The ratio of acetyl-CoA/CoASH is therefore a determinant of PDH activity (12, 13, 15, 42, 43, 44, 45, 46, 47). However, we found that insulin induced an increase in both cardiac acetyl-CoA and CoASH content with no change in acetyl-CoA/CoASH. Thus, the requirement for ACC-dependent production of malonyl-CoA for insulin-induced activation of PDH does not appear to be driven by a change in acetyl-CoA/CoASH. Measurement of total tissue acetyl-CoA and CoASH content does not, however, account for potential changes in mitochondrial acetyl-CoA/CoASH. If acetyl-CoA is found to increase in the cytosol but decrease in the mitochondria along with an increase in CoASH then a drop in acetyl-CoA/CoASH could be responsible for the reciprocal regulation of fatty acid oxidation and PDH activity. For example, insulin-induced increases in malonyl-CoA and inhibition of CPT1 might trigger the conversion of acetyl-carnitine by carnitine acyltransferase or mitochondrial-derived citrate by ATP-citrate lyase to acetyl-CoA in the cytosol (20, 48, 49). It is difficult to accurately assess the subcellular distribution of acetyl-CoA and CoASH due to rapid changes that occur during fractionation. Future studies must therefore employ approaches to measure acetyl-CoA and CoASH levels within specific intracellular compartments and trace the origin of these metabolites. Finally, it is important to note that insulin promotes dephosphorylation of PDH, with the relative level of dephosphorylation of Ser^232^ (site 3) greater than Ser^293^ (site 1) and Ser^300^ (site 2). While PDKs have received considerable research attention, far less is known regarding the regulation of PDPs. Studies with purified preparations from different tissues indicate that PDP1 has ∼ 3 to 6-fold higher rates of dephosphorylation of all sites compared to PDP2, with both PDPs exhibiting greater rates of site 2 dephosphorylation than sites 1 and 3 (50). However, it is difficult to draw firm conclusions on the relative contributions of PDPs to insulin-dependent PDH dephosphorylation *in vivo*. PDP1 and 2 selectivity/activity are likely regulated by multiple metabolic and microenvironmental factors. As such, the potential impact of insulin and inhibition of β-oxidation on PDP activities must be investigated.

Insulin resistance is a common feature of metabolic disorders such as obesity and type 2 diabetes that have been linked to the pathogenesis of heart failure (3, 4, 5, 6, 7). In the heart, insulin resistance has been viewed primarily from the perspective of defective glucose metabolism caused by diminished insulin-stimulated glucose uptake. Our studies highlight the need to consider the interdependence between insulin-dependent inhibition of fatty acid oxidation and activation of PDH when exploring mechanisms responsible for insulin resistance and diminished glucose oxidation. Diminished insulin-stimulated glucose oxidation may involve deficits in malonyl-CoA production, the capacity of malonyl-CoA to inhibit CPT1, and/or the responsiveness of PDH to inhibition of β-oxidation. The molecular mechanisms by which insulin stimulates malonyl-CoA production and subsequently activates PDH must be elucidated. This information is necessary to comprehensively track the temporal development of insulin resistance, identify mechanisms responsible, and tailor specific treatments of cardio-metabolic disorders associated with diminished glucose oxidation. It is important to note, however, that we investigated the acute effects of insulin. Insulin has both short- and long-term effects and caution must therefore be exercised in extrapolating our findings to disease progression.

## Experimental procedures

### Mice

The study employed male C57BL/6N mice and ACC1/2 knockout mice (10–12 weeks of age). Inducible cardiomyocyte-specific ACC1/2 knockout mice were generated by crossing *acaca*^f/f^/*acacb*^f/f^ (51) and αMHC-MerCreMer mice. ACC1/2 knockout was achieved by administration of tamoxifen (3 injections of 0.5 mg in 10% v/v ethanol/sesame oil every other day). The efficiency of ACC1/2 knockout was evaluated by Western blot analysis of heart homogenate. For experiments performed at 12 PM and 12 AM, control (*Acc1*^f/f^/*Acc2*^f/f^) *versus* ACC1/2 knockout (αMHC-MerCreMer x *Acc1*^f/f^/*Acc2*^f/f^) mice were euthanized by cervical dislocation 3 days after the final tamoxifen injection at the respective times of day and hearts rapidly excised and then snap frozen in liquid nitrogen. All procedures were approved by the University of Texas Southwestern Medical Center Animal Care and Use Committee.

### In vivo administration of insulin and/or inhibitors

Mice were injected intraperitoneally with insulin (0.05 U/g) at 8 AM, a time of day when, in nocturnal animals, cardiac PDH is near the daily minimum in activity yet still responsive to stimulation by insulin. After indicated times post-insulin, mice were euthanized by cervical dislocation, and hearts were rapidly excised for immediate preparation of cardiac mitochondria or snap frozen in liquid nitrogen. In experiments that tested the impact of pharmacologic inhibition of ACC, mice were gavaged with 0.5% methylcellulose or the ACC inhibitor, CP-640186 (29, 30) (Millipore Sigma, 100 mg/kg in 0.5% methylcellulose) at 7:30 AM, 30 min prior to injection of insulin. To inhibit β-oxidation in the absence of insulin, C57BL/6N mice were injected with the carnitine palmitoyltransferase I inhibitor, oxfenicine (28) (0.15 mg/g i.p.), at 12 PM, a time of day when hearts of nocturnal animals rely primarily on β-oxidation for ATP production (52) and PDH exists primarily in the inactive form.

### Isolation of cardiac mitochondria

Immediately following euthanasia, hearts were removed and homogenized in ice-cold isolation buffer (10 mM MOPS, 1.0 mM EDTA, 210 mM mannitol, 70 mM sucrose, 20 mM NaF, and 5 mM dichloroacetate at pH 7.4) using a Polytron homogenizer. The homogenate was centrifuged at 500*g* for 5 min (4 °C) and the supernatant (S1) was filtered through cheese cloth. The mitochondrial pellet was obtained by centrifugation of S1 at 12,000*g* for 10 min (4 °C) (40, 41, 53). Mitochondria were resuspended in homogenization buffer to a final concentration of 20 to 30 mg/ml. Protein concentration was determined using the bicinchoninic acid (BCA) method (Pierce) with BSA as a standard.

### Evaluation of PDH activity

Mitochondria were diluted to 0.05 mg/ml in a buffer containing 25 mM MOPS and 0.05% Triton X-100 at pH 7.4. Solubilization of mitochondria with 0.05% Triton X-100 inhibits complex I of the respiratory chain preventing consumption of NADH. PDH activity was measured spectrophotometrically (Agilent, 8452A) as the rate of NAD^+^ reduction to NADH (340 nm, ε = 6200 M^−1^ cm^−1^) upon the addition of 2.5 mM pyruvate, 0.1 mM CoASH, 0.2 mM thiamine pyrophosphate, 1.0 mM NAD^+^, and 5.0 mM MgCl_2_ at pH 7.4 (40, 41, 53). In certain experiments, PDH activity was evaluated in heart tissue homogenate. For these analyses, snap-frozen hearts were crushed over liquid nitrogen. Crushed frozen heart tissue (approximately 10 mg) was suspended in ice-cold buffer (25 mM MOPS, 10 mM EDTA, 5 mM NaF) with brief sonication (3 × 3 s pulses) and diluted in 25 mM MOPS, 0.05% Triton X-100, and 50 mM sodium oxamate (to inhibit NADH consumption by complex I and lactate dehydrogenase, respectively), pH 7.4 (1–2 mg/ml protein). PDH activity was assayed spectrophotometrically (Agilent, 8452A) as the rate of NAD^+^ reduction to NADH (340 nm, ε = 6200 M^−1^ cm^−1^) upon the addition of 0.5 mM pyruvate, 0.1 mM CoASH, 0.2 mM thiamine pyrophosphate, 1.0 mM NAD^+^, and 1.0 mM MgCl_2_, pH 7.4.

### Western blot analysis

Anti-PDH-E1α (ab168379) and anti-phospho-PDH-E1α (pSer^232^ AP1063, pSer^293^ AP1062, pSer^300^ AP1064) were purchased from Abcam and EMD Millipore, respectively. Anti-Akt (4691S), anti-phospho-Akt (Thr^308^ 2965S), Anti-ACC (3676S) and anti-phospho-ACC (Ser^79/212^ 3661S) were purchased from Cell Signaling. Anti-lipoic acid was produced by Covance (Denver, PA), as previously reported (54). The specificity of antibody binding was validated by Western blot detection of protein at the appropriate molecular weight (Precision Plus Dual Color Standards, Bio-Rad, 1610374) and the requirement of primary antibody for secondary antibody binding. For analysis of total cardiac proteins, snap frozen heart tissue was crushed over liquid nitrogen and extracted in T-PER or RIPA lysis buffers containing Halt protease and phosphatase inhibitor cocktail (Thermo Scientific, 1861280). For analysis of mitochondrial or S1 proteins, mitochondria or S1 prepared in isolation buffer containing 20 mM NaF and 1 mM dichloroacetate were suspended in Laemmli sample buffer (Bio-Rad, 1610747) containing protease inhibitor cocktail (Sigma-Aldrich, P2714). Proteins (approximately 6–12 μg/lane, within the linear range) were resolved using 4 to 20% SDS-PAGE gradient gels (Bio-Rad, 4568096) and transferred to nitrocellulose membranes (0.2 μm). Membranes were incubated with primary antibodies at optimized dilution, washed, and then incubated with fluorescent secondary antibodies (LI-COR, 925-32211). Proteins of interest were visualized and quantified using an Odyssey scanner and Image Studio Lite software (LI-COR). To reevaluate given samples for different proteins or phosphorylation sites, membranes were stripped using NewBlot Nitro stripping buffer (LI-COR, 928-40030), checked for complete absence of prior signal, and then reprobed and reprocessed with appropriate primary and secondary antibodies.

### Measurement of nucleotides and short-chain Acyl-CoAs by LC-MS/MS

Metabolites were extracted from snap-frozen cardiac tissue crushed over liquid nitrogen as previously described (55, 56). Briefly, approximately 30 to 50 mg of crushed frozen tissue was homogenized in ice-cold 0.4 M perchloric acid, 0.5 mM EGTA. After 10 min on ice, proteins were pelleted by centrifugation at 21,000*g*. Protein pellets were resuspended in 150 mM KOH and protein content was determined using the BCA assay. Supernatants were neutralized (0.5 M K_2_CO_3_) and precipitates were removed by centrifugation at 14,000*g*. Analysis was done on an API 3200 triple quadrupole LC-MS/MS mass spectrometer (Applied Biosystems/Sciex Instruments) in positive electrospray ionization mode. The mass spectrometer was equipped with a Shimadzu LC-20AD liquid chromatograph and a SIL-20ACHT autosampler. A reverse phase C18 column (Waters XBridge, 150 × 2.1 mm, 3 μm) was used with LC mobile phase consisting of water/methanol (95:5, v/v) with 4 mM dibutyl amine acetate (eluent A) and acetonitrile/H_2_O (75:25, v/v) (eluent B). The gradient increased from 0% to 50% B over 11 min and held for 4 min. The column was washed with 100% B for 4 min and then equilibrated with 100% A for 5 min between injections. The flow rate was 0.18 ml/min and the column was maintained 25 °C. Multiple reaction monitoring measurements were carried out for the detection of the nucleotides and short-chain acyl-CoAs in standard solutions and biological samples. Metabolite content is expressed as nmol/mg protein.

### Metabolite analysis by GC-MS

Cardiac pyruvate content was quantified by GC-MS as previously described (57). Briefly, approximately 50 mg of crushed frozen tissue was homogenized in ice-cold 80% methanol containing an internal norleucine standard. After 10 min at 4 °C, samples were centrifuged (21,000*g*, 4 °C, 15 min) and the supernatant evaporated under N_2_. Samples were incubated in 1% w/v methoxylamine hydrochloride in pyridine (37 °C, 1.5 h) and silylated using N-(t-butyldimethylsilyl)-N-methyltrifluoroacetamide reagent (60 °C, 1 h). The reaction mixture was centrifuged (room temperature, 10 min), and an aliquot of supernatant was analyzed by GC-MS in SIM mode.

### Metabolite analysis by HPLC-UV/Vis

Metabolites were extracted from snap-frozen cardiac tissue crushed over liquid nitrogen. Briefly, approximately 30 to 50 mg of crushed frozen tissue was suspended in ice-cold 0.35 M perchloric acid, 10 mM DTT by sonication. After 10 min on ice, proteins were pelleted by centrifugation at 16,000*g* for 10 min and supernatants were filtered (0.45 μm PVDF). Protein pellets were resuspended in 150 mM KOH and protein content was determined using the BCA assay. CoASH, acetyl-CoA, and malonyl-CoA in the supernatants (100 μl, equivalent to approximately 0.3–0.5 mg protein) were resolved by ion-pair reverse phase HPLC (Waters XBridge C18 column, 150 × 4.5 mm, 5 μm) and detected by *UV/Vis* absorbance at 254 nm (Shimadzu LC-20AD HPLC system equipped with SIL-20A autosampler and photodiode array M20A *UV/Vis* detector). The mobile phase consisted of acetonitrile vs. 100 mM KH_2_PO_4_, 1 mM tetrabutylammonium sulfate, pH 6 using the following elution method with a flow rate of 1 ml/min: 0 to 10 min, 0 to 15% acetonitrile; 10 to 15 min, 15% acetonitrile; 15 to 17 min, 15 to 20% acetonitrile; 17 to 25 min, 20% acetonitrile; 25 to 30 min, 0% acetonitrile. Metabolites were quantified using standard curves constructed with known concentrations of CoASH, acetyl-CoA, and malonyl-CoA (Sigma). Metabolite content is expressed as nmol/mg protein.

### Statistical analyses

Values are represented as mean ± standard deviation. Statistical comparisons were evaluated using the student’s *t* test or in experiments with multiple variables by ANOVA with the Tukey test to obtain indicated *p* values, with *p* ≤ 0.05 considered a statistically significant difference.

### Ethics statement

All procedures involving mice were approved by the University of Texas Southwestern Medical Center Animal Care and Use Committee.

## Data availability

All data are contained within the manuscript.

## Supporting information

This article contains supporting information.

## Conflict of interest

The authors declare that they have no conflicts of interest with the contents of this article.

## Figures and Tables

**Supplemental Figure S4:**