# Supplementary material for: Reciprocal regulation of cardiac β-oxidation and pyruvate dehydrogenase by insulin
Source: J Biol Chem. 2024 May 23;300(7):107412. doi: 10.1016/j.jbc.2024.107412 (PMC11231754; doi:10.1016/j.jbc.2024.107412)
Supplement: Supplemental Figures S1–S4 Legends [file mmc1.docx]

**SUPPLEMENTARY FIGURE LEGENDS**

**Figure S1. Constitutive and Inducible Cardiomyocyte Specific Acetyl-CoA Carboxylase-1/2 Knockout Results in an Increase in Pyruvate Dehydrogenase Activity.** Hearts were harvested from control and constitutive ACC-1/2 cardiomyocyte specific (αMHC-Cre crossed with *Acc1*^f/f^ */ Acc2*^f/f^) knockout mice (10 weeks of age), homogenized, and **A)** Total cardiac ACC1/2 protein content was quantified by Western blot followed by densitometric analysis (n = 4) and **B)** PDH activity was measured in isolated heart mitochondria (+DCA and NaF) spectrophotometrically (n = 5). Hearts were harvested from inducible ACC1/2 cardiomyocyte specific (αMHC-MerCreMer crossed with *Acc1*^f/f^ */ Acc2*^f/f^) knockout mice 4 weeks post-tamoxifen and **C)** Total cardiac ACC1/2 protein content was quantified by Western blot followed by densitometric analysis (n = 6) and **D)** PDH activity was measured in isolated heart mitochondria (+DCA and NaF) spectrophotometrically (n = 8 to 10). Values are presented as the mean ± SD where significant differences (2-tailed *t* test) are indicated by ** *p* < 0.01 and *** *p* < 0.001. Each data point indicates a separate animal.

**Figure S2. Insulin-Dependent AKT Phosphorylation is not Impacted by inhibition of ACC.** C57BL/6N mice were administered the ACC inhibitor, CP 640186 (100 µg/g in 0.5% methyl cellulose) or 0.5% methyl cellulose by gavage (± CP). As indicated, saline or insulin (0.05 U/g body weight) was administered intraperitoneally 30 min later (8 AM). After 10 min, hearts were excised, homogenized, and evaluated for total AKT and phosphorylation status of the Thr ^308^ site on AKT by Western blot followed by densitometric analysis. Values are presented as the mean ± SD (n = 3) where significant differences (ANOVA with the Tukey test) are indicated by *** *p* < 0.001. Each data point indicates a separate animal.

**Figure S3. Insulin-Dependent AKT Phosphorylation is Elevated During the Active Phase.** Hearts were excised and snap frozen from male C57BL/6N mice at 12 PM or 12 AM as indicated. Hearts were homogenized and centrifuged (500 x *g*, 5 min, 4˚C) and the supernatant (S1) evaluated for total AKT and phosphorylation status of the Thr ^308^ site on AKT by Western blot followed by densitometric analysis (n = 5). Values are presented as the mean ± SD where significant differences (2-tailed *t* test) are indicated by ** *p* < 0.01. Each data point indicates a separate animal.

**Figure S4. Injection of Mice with Insulin Does Not Alter the Phosphorylation Status of Acetyl-CoA Carboxylase.** Male C57BL/6N mice received an intraperitoneal injection of saline or insulin (0.05 U/g body weight) at 8 AM. Hearts were excised 10 min post-injection, homogenized, and evaluated for total ACC and phosphorylation status of the Ser^79/212^ site on ACC by Western blot followed by densitometric analysis (n = 3 to 4). Values are presented as the mean ± SD (2-tailed *t* test, no significant difference). Each data point indicates a separate animal.
